# Real-time electronic drug monitoring: experiences from a multinational phase 3 clinical trial to prevent multidrug-resistant TB

**DOI:** 10.5588/ijtldopen.25.0702

**Published:** 2026-05-11

**Authors:** T. Jimah, J.E. Haberer, K.R. Amico, J. Tinkamanyire, M.A. Kendall, S. Moonsamy, R.T. Khan, K.B.F. Fetalvero, N. Suryavanshi, A. Gupta, A.C. Hesseling, S. Swindells, G. Churchyard

**Affiliations:** 1 Bouvé College of Health Sciences, Northeastern University, Boston, MA, USA;; 2 Center for Global Health, Massachusetts General Hospital, Boston, MA, USA;; 3 Department of Medicine, Harvard Medical School, Boston, MA, USA;; 4 School of Public Health, University of Michigan, Ann Arbor, MI, USA;; 5 Aurum Institute PLC, Johannesburg, South Africa;; 6 Center for Biostatistics in AIDS Research, Harvard T.H. Chan School of Public Health, Boston, MA, USA;; 7 Perinatal HIV Research Unit, University of the Witwatersrand, Johannesburg, South Africa;; 8 Y.R. Gaitonde Centre for AIDS Research and Education, Chennai, India;; 9 TB HIV Innovations and Clinical Research Foundation Corp. Silang, Cavite, Philippines;; 10 B.J. Government medical College-CRS, Pune, India;; 11 Division of Infectious Diseases, Johns Hopkins University School of Medicine, Baltimore, MD, USA;; 12 Department of Paediatrics and Child Health, Desmond Tutu TB Centre, Stellenbosch University, Cape Town, South Africa;; 13 University of Nebraska Medical Center, Omaha, NE, USA;; 14 Aurum Institute NPC, Parktown, South Africa;; 15 School of Public Health, University of Witwatersrand, Johannesburg, South Africa;; 16 Department of Medicine, Vanderbilt University, Nashville, TN, USA.

**Keywords:** tuberculosis, TB prevention, treatment adherence, digital monitoring, implementation process

## Abstract

**OBJECTIVE:**

We describe the experience of implementing real-time electronic drug monitoring (EDM) within a clinical trial to prevent multidrug-resistant TB (MDR-TB) among high-risk household contacts.

**METHODS:**

The Protecting Households On Exposure to Newly Diagnosed Index Multidrug-Resistant Tuberculosis Patients (PHOENIx) trial is a phase 3, open-label, cluster-randomised clinical trial conducted in 31 sites in Africa, Asia, and Latin America. Household contacts of adults with MDR-TB were enrolled from June 2019 to January 2025, each receiving an EDM device to monitor adherence to TB preventive therapy. Here, we review selection of the adherence monitoring approach and key procedures implemented to support EDM use and oversight throughout the trial.

**RESULTS:**

Integrating EDM in the PHOENIx trial enabled objective monitoring and early non-adherence identification to ensure timely counselling. Dedicated effort was needed to develop, train, and implement standardised protocols, but overall coordination required relatively few staff. Technical issues were manageable, and monitoring costs were relatively low for a large trial.

**CONCLUSION:**

Findings highlight the added value of objective adherence monitoring, which can inform future trials, particularly for prevention. Our experiences suggest that with the appropriate support, EDM can be used to monitor and characterise adherence, even in a complex multi-country phase 3 trial in settings with a high TB burden.

TB preventive therapy (TPT) is crucial for global TB control, particularly for populations at increased risk of exposure (e.g., household contacts [HHCs] of individuals with TB and individuals with immunosuppressive conditions such as HIV).^1,2^ TPT reduces the risk of TB; however, challenges remain in effectiveness largely due to low rates of initiation, adherence, and completion.^3^ Adherence is a multidimensional phenomenon influenced by patient-level, disease-related, and treatment-specific factors.^4^ Comprehensive assessment is essential to effectively address barriers to adherence, particularly in preventive therapy trials involving asymptomatic individuals who may need additional motivation to sustain long-term adherence to the regimen. Moreover, low adherence may undermine drug efficacy in clinical trials, as biological efficacy depends on actual drug exposure.^5^ For example, at 60% adherence, observed trial outcomes may reflect less than half of the drug’s actual therapeutic benefit.^6^ Adherence monitoring in clinical trials thus enhances confidence in trial outcomes,^7^ yet there is no gold standard approach for measuring adherence,^8,9^ and little has been reported on the processes and considerations involved in assessing adherence in TPT trials. Notably, two randomised prevention trials, V-QUIN and TB-CHAMP, have evaluated levofloxacin for HHCs of adults with multidrug-resistant TB (MDR-TB).^10,11^ MDR-TB was defined as incident, microbiologically confirmed TB with resistance to at least rifampicin and started on treatment within the past 90 days in V-QUIN^10^ and isoniazid and rifampicin diagnosed within the past 6 months in TB-CHAMP.^11^ The V-QUIN and TB-CHAMP trials tested levofloxacin efficacy in preventing TB among HHCs, using pill counts and treatment cards to monitor adherence. The ongoing Protecting Households On Exposure to Newly Diagnosed Index Multidrug-Resistant Tuberculosis Patients (PHOENIx MDR-TB) trial^12^ is taking a different approach by integrating an electronic adherence monitoring strategy to monitor adherence behaviour in HHCs more objectively than traditional methods and to facilitate timely adherence support.^13^ Below, we describe the PHOENIx MDR-TB trial and practical lessons learned from integrating electronic drug monitoring (EDM) within the trial.

## OVERVIEW OF THE PHOENIx TRIAL

PHOENIx MDR-TB trial is a phase 3 open-label, multicentre, international trial conducted by the Advancing Clinical Therapeutics Globally (ACTG) and International Maternal Pediatric Adolescent AIDS Clinical Trials (IMPAACT) networks. This trial is registered with ClinicalTrials.gov, number NCT03568383. The objective of this trial is to evaluate the efficacy of TPT in reducing the risk of developing TB by comparing 6 months of daily delamanid (intervention arm; 200 mg in adults and weight-based dosing for children) versus daily isoniazid (control arm; 300 mg in adults and weight-based dosing in children, plus pyridoxine) in high-risk HHCs of adult index cases with documented MDR-TB. The study is taking place in high-burden settings across 31 sites in Africa (53%), Asia (24%), and Latin America (23%). MDR-TB is defined as resistance to isoniazid and rifampicin and treatment initiation within the prior 90 days. While the trial included both index cases and their HHCs, here we focus on HHCs. Eligible HHCs included children under 5 years old, and individuals 5 years and older with HIV and/or with a positive tuberculin skin test and/or interferon-gamma release assay (IGRA) result. HHC enrolment began in June 2019 and was completed in January 2025. Participants were offered study drug for 26 weeks and followed up for a total of 96 weeks.

At study enrolment, HHCs received an EDM device (i.e., a ‘smart’ pill container with an electronic insert that records each opening as a proxy for medication ingestion and transmits a time-and-date stamp of the opening to a central server via cellular networks, Figure) with monthly medication refills. However, lack of pill ingestion despite device opening cannot be detected and is a limitation of the measure. If multiple individuals were enrolled within a given household, each received their own labelled EDM device. Functionality checks were conducted monthly, and educational sessions on EDM covered appropriate use of the device and tailored adherence support at enrolment and as needed throughout the trial. Sites monitored adherence data and received alerts for participants with three or more doses missed during the preceding 7 days.^14^ Study site staff then investigated any technical challenges and/or provided adherence support as needed using a context-specific tiered approach (e.g., initial contact through text messaging or live phone calls, followed by a home visit if needed).

**Figure.**
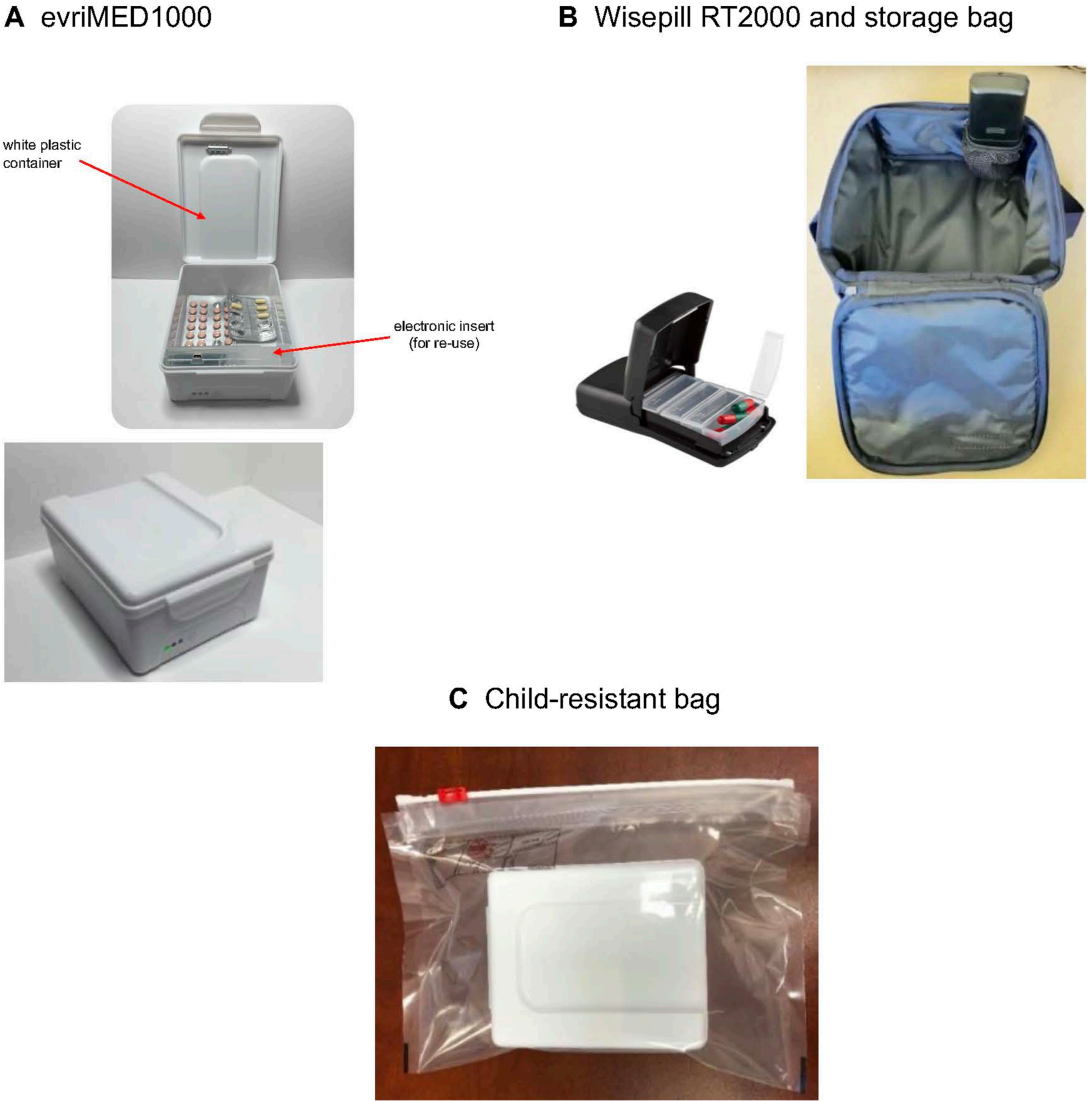
Electronic drug monitoring Wisepill devices for storing medication. **A**:evriMED1000 was used in all sites except Thailand, which due to cellular network infrastructure used (**B**) RT2000; **C:** indicates the use of a child-resistant bag for safety purposes. Images were provided courtesy of Wisepill Technologies.

### Ethical statement

The study protocol and informed consent forms were reviewed and approved by each site’s local institutional review board/ethics committee (IRB/EC) and any other applicable regulatory entity. Informed consent (and assent, where applicable) was obtained from all study participants.

## IMPLEMENTING REAL-TIME ELECTRONIC DRUG MONITORING

Selection of an appropriate tool for monitoring adherence was an iterative process involving comparison of available approaches (Table 1). The study team considered different real-time and standard electronic monitoring devices, clinic-based and random pill counts, self-report approaches, and pharmacokinetic measures (e.g., drug concentrations in urine, hair, and blood).^8,13,16–18^ Device selection prioritised real-time non-adherence detection and support, cost, functionality, and acceptability among global populations. The Wisepill device (Wisepill Technologies, South Africa) was selected for its ability to record daily adherence patterns. EDM provides a more objective assessment and minimises social desirability and recall biases, compared to self-reports and pill counts.^13^ Wisepill technology records device openings in real-time, which are used as a proxy measure of medication-taking behaviour.^8,13,19^ Monitoring is continuous with alarms for participant-chosen dosing times, thus enabling early detection of non-adherence and timely participant support. Studies have demonstrated Wisepill’s effectiveness and technical feasibility in low-resource settings,^20–23^ including high correlation with pharmacokinetic measures in monitoring HIV pre-exposure prophylaxis (PrEP) adherence in Kenya.^24^

**Table 1.** A comparison of different adherence metrics.

Adherence measure	Description	Merits	Potential limitations
Electronic adherence monitors	Electronic pill dispensers that track and log when devices are opened	- Can record data in real time	- Require technical infrastructure (cellular access, electricity, charging)
		- Convenient for individuals as medication can be taken remotely	- May be expensive to implement and maintain given the cost of devices, shipping, and hosting service
		- Can provide adherence analytics and be programmed to detect adherence thresholds with potential for intervention	- Can be considered bulky, inconvenient for travel, and stigmatising
		- Can provide timely reminders to support medication intake via SMS or visual alarm system	- Cannot confirm actual ingestion
		- Some devices have shown feasibility in low-resource settings	- Within households, devices could be confused among participants
Mobile applications and video recording	Smartphone applications with recording verification of medication intake	- Convenient for the individual as medication can be taken remotely	- High initial costs and require investment in hardware (phones, servers) and skilled personnel for coding and app development
		- Can provide adherence analytics with potential for intervention	- Can be burdensome and time-consuming for individuals to record themselves, as well as raise concerns about privacy and confidentiality
		- Can record ingestion	
Pill counts	Manual counting of remaining pills	- Do not require extensive training to implement	- Can be labour-intensive
		- Low cost	- Prone to human error
		- Familiar in clinical and research settings	- Cannot confirm actual ingestion
			- Do not allow for real-time intervention deployment
Self-report	Patient-reported information about their own medication-taking behaviour	- Cost-effective due to minimal infrastructure	- Prone to recall bias
			- Prone to social desirability bias
		- Can be easily integrated into routine care	- Cannot confirm actual ingestion
			- Limited to age range appropriate for self-report
Pharmacokinetics	Measuring drug concentrations in blood/urine/hair to confirm medication intake	- Can objectively assess adherence	- Can be costly to implement due to reliance on laboratory testing and specialised equipment
			- May not reflect true adherence due to whitecoat dosing behaviour^15^
			- Reference values are not clearly defined for all medications

The study team also considered implications of EDM for anticipated US Federal Drug Administration review of the findings of this phase 3 trial. Specifically, concerns were raised that EDM would need to be included in approval of the study drug, should it be proven effective. Ultimately, the team determined EDM would be presented as a tool for more accurate assessment of efficacy. Future phase 4 studies could then explore the role of adherence monitoring and support on effectiveness in practice, as with other prevention drugs, as was done with PrEP.^25–27^

### Use of the Wisepill device

Among multiple Wisepill devices, evriMED1000 (Figure) was selected because of its relatively low cost and size that accommodated isoniazid and delamanid blister packs. The device provides audible dosing reminders and operates on all networks including the G2 networks available in most participating countries. In Thailand, the RT2000 version was used for G3 network compatibility. Universal SIM cards enabled multi-country participation. The EDM device (i.e., electronic insert) was reused among HHCs at each site, with unique containers provided to each participant to avoid pharmaceutical contamination. Devices were placed in child-resistant bags for safety purposes. Study data were hosted by Wisepill Technologies in South Africa, with sites accessing unique database logins. The EDM device registers a daily ‘heartbeat’ even if the device was not opened to confirm device functionality and distinguish between device openings and malfunction. HHCs brought the devices to every study visit for pill refill, battery swap/charge, device functionality checks, and repair as needed. To enable appropriate follow-up, we needed to determine a threshold value for triggering non-adherence. Based on TB treatment modelling studies^14,28^ and investigator consensus, three missed doses over a running 7-day period was selected as indicative of risk for a lack of therapeutic effect. Sites contacted HHCs through appropriate channels to assess device use and medication adherence non-judgmentally. Device heartbeat helped identify technical issues requiring troubleshooting rather than counselling; technical support was available through the study and Wisepill Technologies.

### EDM oversight and training for study site staff

Adherence monitoring was led by two senior researchers experienced in adherence measurement and counselling, supported by a data engineer and Wisepill Technologies. An adherence working group included trial principal investigators, study medical officers, ACTG and IMPAACT representatives, and the data centre. With 31 study sites across 13 countries, EDM implementation was highly standardised with central oversight. The study protocol detailed adherence procedures in the manual of procedures (Supplementary Data), covering device setup and labelling, device education and regimen planning, adherence monitoring and support, functionality assessment, dose reporting, and notification of study completion. Sites received EDM training through manuals and sessions conducted individually or in groups. The adherence experts initially provided in-person regional trainings to clusters of study sites, later transitioning to multisite webinars that were found effective and continued during the COVID-19 pandemic. Refresher trainings were provided periodically, covering technical aspects and adherence challenges (25 total training sessions).

Standard operating procedures addressed potential EDM device limitations,^13^ including proper opening/closing of the device and minimising device non-use (e.g., pocket dosing). Study site staff were trained to review monthly EDM data with HHCs. Sites underwent a run-in review period after enrolment completion through the 10th household randomisation. Technical challenges were also addressed through rapid process improvement methodology for up to 6 months, which established practical guidelines for local sites to enhance participant adoption of the EDM device. Tracking spreadsheets were provided to study site staff to document issues which could then be shared with the Wisepill support team. The adherence working group reviewed data bimonthly and provided feedback to foster discussions with HHCs. Remote support was provided to study site staff via video conferences to address sites with notable or persistent non-adherence episodes and as requested.

### Participant education and counselling

When HHCs received EDM devices, study site staff provided education and device training. Study site staff explained the importance of adherence, discussed factors that may influence adherence, and identified ways household members could support each other in daily dosing. HHCs were advised to limit EDM device openings for pill-taking and to avoid device swapping. Labels were created for each device to avoid confusion among individuals within a single household. Sites were also encouraged to use local identifiers like colourful stickers for children’s devices. Plans were encouraged for travel or other situations in which the device may be challenging to use (e.g., due to logistical issues and perceived stigma). HHCs received visual feedback on their dosing patterns between visits as part of the adherence support and counselling sessions.

### Cost of EDM devices

While a formal costing analysis of adherence monitoring was not undertaken, the primary estimated cost of implementing EDM in the study is detailed in Table 2. The total cost of $371,266 includes $160,930 for the purchase of 2,144 EDM devices (to improve cost-efficiency, electronic inserts [Figure panel A] were reused, while each participant received a separate container to avoid any product contamination) and accessories such as multi-battery chargers and spare batteries; $170,336 for SIM card data, web hosting services, and international shipping; and $40,000 for personnel costs in EDM training and supervision. With 3,905 HHCs, the average cost per participant was approximately $95.

**Table 2.** Measured electronic drug monitoring (EDM)-related expenses^A^.

Item	Description	Number purchased	Cost per item (USD)
EDM kits (2G, 3G, LTE)	evriMED1000C EDM device (including SIM, battery, and container), Wisebag, device, charger, USB cable	2,144	EDM kits (2G – $50.00; 3G – $200.00; LTE – $175.00)
Medication containers	evriMED reused medication containers	2,436	$5.00
	Multi-chargers	58	$75.00
	Spare batteries	270	$10.00
Service	SIM data service (per device, per month)	57,093	$1.50
	Web hosting service (per participant, per month)	26,987	$2.00
	SMS (text) bundles (each SMS sent)	32,000	$0.05

A Additional unmeasured costs include the costs of shipping items to study sites and compensation for study site personnel. Note that sites generally incorporated EDM-related activities within existing staff roles.

### Personnel costs in EDM training and supervision

The only roles dedicated solely to adherence monitoring were 5% of one senior researcher’s time and an approximate yearly salary of USD $30,000 for the data engineer. Contributions from other study investigators leveraged their other roles in the study. Each study site took a customised approach to implementing the adherence monitoring and support protocols; however, all sites utilised existing staff roles, including site pharmacists, study coordinators, and counsellors. Support costs from Wisepill Technologies were included in the equipment and hosting fees (Table 2). Although not discretely funded, the statistical and data management team developed a tailored analytical approach for efficient processing and analyses given the substantial volume of data collected.

### Overview of EDM challenges and associated responses

The primary challenges in EDM included technical problems, human-related factors, and logistical issues as detailed in Table 3. Technical problems involved poor network coverage affecting data transmission, battery issues, and firmware/server bugs early in the study. Human-related factors impacted accurate data capture, including incorrect EDM electronic insert positioning in the pill containers (Figure), improper device opening/closing, and device non-use. Curiosity openings (i.e., when individuals open the device, not for dosing, but simply out of curiosity or to explore device functionality) and pocket dosing (i.e., a single opening event where multiple doses are removed and placed in an alternative container) led to over- and under-estimation, respectively. Logistical issues including changes in customs regulations during the COVID-19 pandemic lockdown caused delays in device delivery. Solutions to these challenges involved reconfiguring device settings, replacing batteries and SIM cards, updating firmware, and proactive shipment ordering. Site teams reinforced proper device usage through counselling and troubleshooting challenges. Multiple device openings in a single day were considered as a single dose for data interpretation. Disruptions due to lockdowns and unrest were minimised through remote monitoring. Technical issues were resolved via phone, while device and/or battery exchanges and medication delivery continued without direct contact.

**Table 3.** Technical problems, human-related factors, and logistical issues associated with electronic drug monitoring in the PHOENIx trial.

Type of challenge	Issues	Solutions implemented by site teams
Technical	- Poor network coverage affecting data transmission	- Reconfigured device setting
	- Battery issues (e.g., low charge, loose fitting, swelling, and factory defects)	- Replaced batteries and SIM cards
	- Firmware/server bugs	- Updated firmware
Human-related	- Incorrect positioning of the sensor device in the pill container causing inaccurate adherence measurements, or improper opening/closing interfering with data capture	- Reinforced proper use and placement of the sensor device in the pill container
	- Size of the pill container and perceived stigma deterred use	- Provided counselling support to mitigate stigma concerns
	- Curiosity openings (i.e., opening the device, not for dosing, but simply out of curiosity or to explore device functionality) overestimated adherence	- Recorded multiple daily openings as a single dose
	- Pocket dosing (i.e., a single opening event where multiple doses are removed and placed in an alternative container) underestimated adherence	- Reinforced use of the pill container for storing medication
Logistical	- COVID-19 lockdowns and political unrest	- Delivered medication without direct contact, including remote monitoring and troubleshooting via phone
	- Changes in customs regulations	- Proactively placed shipment orders for the pill containers in advance

## DISCUSSION

The PHOENIx MDR-TB trial sets a precedent for the implementation and potential benefit of including individual-level electronic drug monitoring in multinational phase 3 clinical trials, including low-resourced settings. Dedicated effort was needed to develop, train, and implement standardised protocols, but overall coordination required relatively few staff. Technical issues were manageable, and monitoring costs were relatively low for a large trial. Our findings suggest that similar adherence strategies could benefit future TB prevention trials.

Poor adherence can significantly impact reliability of trial outcomes by underestimating drug efficacy and undermining interpretation of results, as was seen with the VOICE and FEM-PrEP Trials for HIV prevention.^29,30^ Findings from the VOICE and FEM-PrEP trials, which assessed novel PrEP regimens for HIV prevention among women, indicate that suboptimal adherence likely attenuated drug efficacy and contributed to the lack of reduction in HIV incidence, emphasising the need for more rigorous adherence assessment in future prevention trials.^29,30^ Integrating electronic monitoring in the PHOENIx MDR-TB trial enabled objective monitoring and early non-adherence identification for timely patient counselling, which will increase confidence in data interpretation at the trial’s conclusion. While implementation concerns and related costs of EDM have been reported in previous studies,^31–33^ we demonstrate an EDM implementation approach that should be considered for MDR-TB prevention trials.

Implementation of adherence monitoring varies by context and monitoring objectives,^18,33^ affecting outcomes and highlighting the need to understand factors influencing successful deployment. Within clinical trials, comprehensive monitoring and support jointly help ensure the optimal ability to measure efficacy. In routine clinical care, medication adherence monitoring sometimes occurs without adequate support, becoming an end in itself, to the detriment of adequate patient support.^34^ Use of EDM and other strategies should thus be considered carefully for each implementation context.

The PHOENIx MDR-TB trial demonstrates several strengths for future MDR-TB prevention trials. First, a major strength is its unprecedented scale, encompassing over 5,000 index cases and HHCs across 31 diverse sites worldwide, producing rich data and enhancing the generalisability of findings. Second, the trial offers high-value adherence data at a low cost. Third, electronic adherence monitoring enabled remote real-time tracking of medication intake even amid the challenges posed by COVID-19 lockdowns, which may be relevant for similar future challenges. Adherence data and their impact on trial outcomes are still pending in the PHOENIx MDR-TB trial; specific results cannot be disclosed until trial completion to preserve data integrity and validity in accordance with the study protocol. While monitoring costs were low, no formal cost analysis was conducted; hence, implementation costs may be higher in cases where existing staff roles cannot be leveraged. Overall, experiences from the PHOENIx MDR-TB trial present an adherence monitoring model for future MDR-TB prevention trial investigations in low-resource settings.

## CONCLUSION

Accurate adherence monitoring is essential for reliable evaluation of outcomes of TPT for MDR-TB. Real-time adherence data support participant engagement and ensure appropriate interpretation of trial results by accounting for actual drug exposure. Our experiences suggest that with the appropriate support, EDM can be used to monitor and characterise adherence, even in complex multi-country phase 3 clinical trials in low-resource settings. We anticipate that this nuanced data quantifying patterns of drug exposure will advance our ability to answer primary questions about drug effects on TPT.

## Supplementary Material
