# Supplementary material for: Real-time electronic drug monitoring: experiences from a multinational phase 3 clinical trial to prevent multidrug-resistant TB
Source: IJTLD Open. 2026 May 11;3(5):305–11. doi: 10.5588/ijtldopen.25.0702 (PMC13160272; doi:10.5588/ijtldopen.25.0702)
Supplement: Supplementary file 1 [file ijtldopen25-0702_supplementarydata1.pdf]

SECTION 4: ELECTRONIC DRUG MONITORING (EDM) DEVICE

4.1 TECHNICAL INFORMATION FOR THE SETUP OF DEVICES

Technical information on the setup and management of the EDM devices is available in the Wisepill evriMED and SENSE3.0 Manual and Wisepill RT2000/Wisebag and SENSE3.0 Manual. "EDM device" in the text that follows refers to both the evriMED1000 and the RT2000/Wisebag electronic insert unless otherwise noted.

Table 4.1 -1 Comparison of evn"MED1000 and RT2000/Wisebag

Feature/Function	evriMED1000	RT2000/Wisebag
Mobile Network	2G – Rest of the World	3G-Thailand
Battery	1800mAh LiPo – External charging	1100mAh LiPo – Internal charging
IMEI	15 Digit Number - On the battery door	15 Digit Number- Inside the lid
Serial Number	24 character Alphanumeric	7 character Alphanumeric
Event upload frequency	First event of the day	Real-Time
Intervention/reminder	Audible/Visual Alarm	SMS

Individual login accounts will be created by Wisepill for the study staff. Each site will only be able to view its own devices and will not have access to the information at other sites. There are 2 numbers allocated to each EDM device: IMEI number and the serial number. The IMEI and serial numbers are unique to the EDM device and cannot be changed. The IMEI will be listed as the device on the Wisepill website. A new client must be created on the Wisepill website, SENSE system for every household contact (HHC) and will serve as the Identification Code. The format should be as follows: site number-PIO number (e.g., 11459-12345678). Once a site has received its devices, the devices are only to be used by HHCs from that site. Devices are not to be interchanged between sites. When a device is to be dispensed for the first time, an email should be sent to Wisepill Technologies to request the activation of the device's SIM card. The device IMEI number must be included in this email request. If, for any reason, a device that has been activated is not going to be used for a period of 2 months or longer, the following steps must be followed:

- Send an email to Wisepill Technologies requesting deactivation of that device's SIM card. Include the device IMEI number in the email.
- The battery should be charged for at least an hour before the device is stored in order to prevent the battery from losing all its charge
- If a deactivated EDM device and SIM card are to be used again, another email must be sent to Wisepill Technologies requesting that the SIM card be reactivated.
- It is preferable that EDM devices that have been used before, be used again by reassigning a Client (participant) to the device, instead of activating new devices. Using the End Participation function on the SENSE system, the previously used device will be available to assign to a new Client.
- The white plastic EDM container should not be re-used among HHCs. Rather, the electronic insert should be removed from a used container and transferred to a new container for each HHC.

When an EDM device is not going to be used again (i.e., at the end of the study), an email needs to be sent to Wisepill Technologies requesting deactivation of the device's SIM card. Include the device's IMEI number in the email. To replace a faulty/stolen/misplaced EDM device of an active HHC, the reassign device feature on the HHC's adherence calendar page should be used. Click the "Edit" feature under the "Device assignment" section, select a device with an activated SIM card and click "Assign".

PLEASE NOTE: HHCs SHOULD NOT keep/store the EDM device in a steel cabinet. This disables the device and will prevent the device from communicating with the server and transmitting battery levels or opening event information.

<u>Precautions for sites with poor cellular network coverage:</u>
The EDM device may have trouble connecting to the network to sync with the first opening if the network is poor. By default, the date on a new device is 01-01-2000; this date will also appear every time the battery is disconnected. The first event from the device will be transmitted to the server with the 01-01-2000 date and will not be recognized by the server as a correct date to update the intake field. If this date appears, you may need to disconnect and re-connect the battery several times, which can delay enrollments. This situation can be mitigated if the batteries are connected to devices after SIM activation but prior to enrollment, so you have time to confirm an initial sync has occurred. The battery can be disconnected and re-connected just before being given to a HHC to preserve the charge if a long period of time will elapse prior to use.

Syncing alarm for configuration and intake settings:
At the end of each session, when a device (with a charged battery) connects to the server, the device will request a configuration update from the server. When the device configuration has been updated, the device will reboot followed by a buzzer sound and quick LED flash, thus indicating that the configuration has changed. This sequence of events will occur every time that the configuration is changed/updated. When this happens, the intake settings on the server should also have been copied down to the device.

4.2 ORDERING OF THE DEVICES

Getting Started – Key order of operations

a. Device accounting: The evriMED or RT2000 electronic inserts and containers will be shipped separately via courier. Unpack and double check the inventory (the list of IMEI & SIM numbers will be sent by Wisepill). Notify Wisepill of any discrepancies.

b. SIM Cards
The SIMs card should already be installed. If the SIM cards are not installed, please contact Wisepill Technologies. Please contact Wisepill Technologies at least 7 days before the anticipated date of enrolment to activate your SIM cards.
Please make sure that the SIM cards are activated before installing the battery.

c. Batteries
Batteries must be charged before installing in the devices. If the batteries were charged and installed before the SIM card was activated, the battery will have to be disconnected and reconnected after the SIM card is activated otherwise the device will not synchronize the correct date and time settings. To avoid delays in receiving the first intake, connect the battery at least 2 minutes after completing the participant registration process (i.e., after clicking Next Step on the SENSE system). The sites do not have to order devices. EDM devices will be shipped to the sites at a time close to site activation, according to enrollment projections provided by the sites. Subsequent shipments of EDM device electronic inserts may be requested depending on the site's enrollment numbers. However, the white plastic EDM container should not be re-used among HHCs. Additional containers for the lifetime of the study need to be ordered by sending a request to Wisepill Technologies. Wisepill typically requires the following items/documents from sites, but each country has its own regulations, which should be discussed with the site coordinator.

- A clear description and quantity of the goods the site is ordering (i.e., EDM electronic inserts, plastic containers, multi chargers etc.).
- A delivery address, contact person (preferable 1 and a maximum of 2) and a contact number.
- Clarify if a donation letter is required (which Wisepill will provide if needed).

4.3. ADHERENCE MONITORING AND SUPPORT

4.3.1 OVERVIEW

Adherence monitoring and support includes use of electronic dose monitoring (RT2000/Wisebag at sites in Thailand and the evriMED1000 at all other sites), HH unit education, regimen planning, outreach to participants for non- adherence episodes (3 out of 7 days without opening the device; see section 4.3.5.3 for additional details), and HH unit review of collected dosing information (dosing graphs from the EDM) at regularly scheduled study visits. This provides the opportunity to offer continuous support at clinic visits and between visits as well.

4.3.2 ISSUING THE EDM DEVICE

At the first issue of the device to a HHC, the following information should, at a minimum, be shared with the HHC and can be done as a group visit if several or all members of the HH are present:

4.3.2.1 Important Information for the HHCs

What is this device?

- This device is used to gather information about dosing/taking a dose of the medication. Each time the device is opened, it records the date and time. If people only open the device when they are taking a dose, this allows for collection of accurate information about dose time that is otherwise very difficult to gather.
- The opening dates and times are communicated automatically through the cell phone network to a protected study website. If the cell network is not working or is out of range, the device will record and store the information until a connection can be made.

When in range or network is restored, the data are communicated.

- The device does not collect other data besides opening dates and times, and whether the device is working (e.g., battery levels).

Special Features of the Device

- Each device will be set to expect an opening at a specific dose time. An alarm will beep around the specific dose time if the device has not been opened. The beeping will stop only when the device is used (i.e., opened for a minimum of 2 seconds and closed).

How is it used?

- The collection of dose times helps because it will describe the situations where the medications may or may not work in relation to taking and not taking the medicines.
- Site staff members will monitor to see if any HHC has a device that is not working right and if the HHC might need some additional help.
- If the device is not functioning properly or an HHC might need some additional help, the site staff may reach out to the HHC to offer assistance or to repair a device.

Things we are asking HHCs to do:

- Each HHC will be given a device that must be clearly labelled so HHCs do not get confused with whose device it is. Refer to section 4.5 for explanations and suggestions regarding the labelling of the device container.
- Only store the study-issued medicine in the device.
- Dose once daily from the device.
- Bring the device with them to study visits.
- Understand that it is acceptable to travel with their device or take it outside the home.
- Once the HHC has stopped taking study drugs, the EDM electronic insert is removed from the container for the site to reuse. The HHC can keep the white plastic container or bag, if desired.

HHC should be instructed NOT to:

- Submerge device in water - it does not need to be cleaned in general and if needed just clean with a damp cloth.
- Drop or hit device - if the device is dropped or an accident of this nature occurs, call the study site.
- Subject to extreme heat or extreme cold.
- Store anything but the study medication in the device.
- Store the device in a steel cabinet.

Things to discuss further with each HHC/HH:

- If more than one HHC is enrolled in the study and has a device, discuss strategies for only using their own specific device.
- HHC should discuss potential times or situations for device non- use (e.g., travel) and how to minimize them, and concerns with stigma.
- If the HHC feels he/she cannot take the EDM device container with them, they should make use of an alternative container and at their next study visit inform the site staff about the period they used another container.
- If rattling of study medication is bothersome, they should use some cotton wool to stop medications from rattling.
- The EDM device is meant to HELP, not to be intrusive or invade privacy. It is how the site staff can stay connected and available to participants between study visits. Spend time discussing this with HHCs and exploring any possible concerns with this.

Practice using the EDM device: The study pharmacist will fill the device with study medication at enrollment (DLM should only be removed from the blister pack at the time of dosing). The process of opening the device and removing medication to prepare to take a dose, will be demonstrated to the HHC by the pharmacist or designee.

4.3.2.2 Important Information for the Site Staff

- If a participant forgets to bring the device to a study visit, the study medication can be dispensed without it and the HHC can fill the container at home.
- Devices should be retrieved from HHCs when they stop taking study drug. However, the HHC may keep the container that held the EDM electronic insert.
- Collect information about dose time - the system will use a daily alarm if the device is not opened on time (i.e., within 4 hours before the set time).
- Opening and closing the container (minimum opening of 2 seconds) to take study medication will cancel the alarm for that day.

  Blinking lights:
    - Green light: Device is working → nothing to do.
    - Yellow light (temporary): Device attempting to sync with errors (network or server) → take note.
    - Red light: Problem → the participant should contact site staff.

4.3.3. EDM DEVICE DISPENSED WITH EDUCATION AND REGIMEN PLANNING

4.3.3.1 Overview: Although individual HHCs have given consent to participate in the study, the education about and subsequent support of adherence should be conducted with all the enrolled HHCs together whenever possible (HH unit). Engaging all HHCs receiving study medication can reduce confusion and harmonize information across the whole household. This can foster support among HHCs, which is ideal for the study. HHCs influence one another and fostering positive interactions around the medication, and support throughout the household can be very valuable. By working with all HHCs, we are trying to foster a team, collaborative approach. There may be times when all HHCs receiving study medication cannot be engaged- maybe the HH unit is fragmented and not a social unit, maybe some participate but not all. Or there may be splits within HH units while on study. In cases when education and support as a group is not possible, an individual focus should be adopted.

4.3.3.2 Education (First and early HH discussions)
Education is the dominant focus at screening, enrollment and first dispensation.

4.3.3.3 Important Information for the HHCs
Site staff members are encouraged to think about the answers to the questions below that may be most common in their area. The basic information that would be potentially important for HH units to discuss is noted below. Note however that these are not intended to be information messages to be provided to all HHC study participants. Rather, they are topics that a facilitator could ask about, use as the topic of an activity, have different HH unit members discuss or debate, and so on. The reason these are NOT to be given as messages is because some, in fact most, may have already been discussed at the time of enrollment or consent. Here the facilitator is hoping to explore or "work with" what HH unit members recall and areas where they may have doubts.

Examples of Question/Concepts to explore:

- What is TB? What is the treatment for TB?
- How common is it for someone to get TB from someone in a household?
- What are the ways people use to prevent getting TB right now (not study-related)?
- What is special about this study's approach?
- Why do they think it would be valuable to look at these particular study medicines?
- How will study researchers check whether the study medicines actually work to prevent TB?
- How would this medication work, if it does work?
- Why is adherence important?
- If the study medication works, when would it be available in THIS community?
- What do I do if I don't want to take this medication after already enrolling?
- How do I know if the medication is safe? What if I hear about something or experience something scary?
- How do I know what goes on with any biological samples I give?

4.3.4 METHODS FOR EXPLORATION OF TOPIC AREAS

- Ask. Present the topic to the HH unit as something others in the study have wondered about. Ask what their understanding is. Ask if they could understand where it could get confusing. Use this as an opportunity to introduce new information.
- Draw. Ask unit member to draw out what TB looks like to them. What TB prevention means to them. Any of the concepts can be drawn out - there is no correct drawing, but it provides an opportunity for members to discuss their beliefs and understanding of important topics. Drawing is appropriate across ages (children and adults can draw!).
- Storytelling. Units can be asked to come up with a short story about someone who was scared of the study medicines and then found out something from someone that made them feel reassured. Other stories can be created about TB prevention and other areas in which trust can be built between the study and the participants.

- Acting. Any story can be acted out. Children can act out being TB and infecting someone or being medicine that protects people from TB (including HHCs who are "being" TB and so on.
- Song/Dance. HH units can create songs or dances around TB, the case getting better/successful TB treatment, or the unit contributing to finding out ways to prevent TB.

Each site should plan how they will work with HH units on education around TB, TB prevention, how study medication might work to prevent TB, and why regular dosing matters.

4.3.4.1 Regimen Planning (First and Subsequent Visits)

After providing education about the study and TB more generally, discussing the specific regimen is part of the first visit and should be revisited at subsequent visits especially if someone is struggling with adherence. Each aspect of regimen planning can be done as a group discussion. Be sure to summarize the approach and specifics
at the end of the discussion. Don't forget to ASK about strategies people will try to use (item (3) below).

REGIMEN PLANNING
Discuss specific plans are for:
(1) placement/storage of EDM for each HHC and how to tell whose is whose
(2) dose times for each HHC (this may have been decided on already- if so, discuss times that were selected)
(3) strategies people plan to use for themselves, children or others in the household

Sites are encouraged to informally ask HHCs about their experience as they begin using the EDM device. Questions from the acceptability interview, which is part of each participant's week 26 visit, may also be used for this informal acceptability assessment. These data can be kept in a local database and concerns should be reported on the EDM device functionality log.

4.3.4.2 Suggestions on how to Facilitate Discussions around Adherence (First and Subsequent Visits)

In addition to the strategies discussed in regimen planning, ask participant(s) to think about when adherence might be challenging and when they would certainly NOT be adherent. Two methods for this are "what if" and "that's enough" situations.

What if?
The "what if" situations can highlight what someone's main concerns are and help to brainstorm ideas for what to do in those situations.
What if I think this is bad for me or my child? What if I feel sick? What if someone tells me I am foolish to be in this study? What if the device seems not to work? Basically, what are the things that come to mind where you really would not know what to do?
That's enough! It can also be helpful to discuss the "that's enough" situations or events. Here the discussion focuses on what would need to happen for someone to decide they will NOT take the medication. This exploration is intended to foster critical decision making. There are always situations when someone would stop. We want people to know that we are aware of that and still want them to be a part of the study even if something has happened that caused them to decide to stop taking the medication. Sometimes participants will tell you there literally is no situation that would have them discontinue.
This should raise some concern because there has to be some situation, no matter how far-fetched, that would cause someone to discontinue the medication or even drop out of the study. Help people to explore the extremes. The point of this is NOT to scare or introduce concerns that are not there, but rather to open the door to talking about intentional non-adherence and how that too can be something to discuss openly. We should not focus only on problem-solving; that assumes doses were missed unintentionally. We want to allow people to discuss intentional avoidance of study medication as well so we can engage in a discussion around building confidence and decreasing skepticism towards study medication, procedures or the study as a whole. Establishing yourself as someone who can be trusted and who understands that blind "compliance" is not the goal. We want participants to be open with us.

4.3.4.3 Face to Face Wrap Up

For first and subsequent sessions - be sure to wrap up the visit by asking about any issues the participant(s) have with any material discussed or instructions. Express sincere thanks for contributions and emphasize open discourse- even if it means talking about dislikes or non-adherence.

EDM DEVICE FUNCTIONALITY CHECK

Dedicated site staff should monitor the EDM devices regularly using the Device and Client Status Report or the Consolidated Activity Report on the Wisepill website; see Wisepill User Manuals to see device information. The Client Event Report will provide a list of the HHCs with low adherence. See the user manual for details on how to configure the reports. Devices are monitored for functionality, battery life and non- adherence episodes.

4.3.5.1 Functionality

Heartbeat: the heartbeat is a daily signal to site staff to indicate that the device is working; heartbeats should be monitored by a site staff member regularly, so that persistent lack of heartbeats (e.g., 3 days) can be identified in between visits and corrective action taken as needed.

4.3.5.2 Battery

Site staff will change the battery at regular study visits, as needed, and batteries should be changed at the site in between study visits in case they unexpectedly run low.

A battery should never run down totally and lose all its charge. This could lead to the battery charger seeing the battery as dysfunctional and not being able to charge the battery.

Important Safety Information

- Any battery showing signs of swelling (puffing up) should be disposed of safely.
- Battery swelling can occur for a few reasons:
  - Over discharging: The Howell battery contains an internal circuit to prevent discharging below 3V. But, if a battery is left in a device after a participant has completed their treatment or if it is stored at a very low voltage, the battery may continue to discharge at a slow rate, and over a prolonged period, it may be compromised. Charging a battery that has been discharged below 3V can lead to swelling.
  - Exposure to a high temperature: This is addressed in the Wisepill Technologies Battery Safety Guidelines V2.3 dated April 2, 2024. The guidelines state that it is best to store batteries in their original containers in a cool place. Do not store the Lithium Polymer pack in extreme temperatures below 0C or above 50C.
  - Overcharging could in theory cause swelling; however, the Howell battery contains an internal circuit that prevents overcharging from happening.
    - The multi-charger charge rates for each of the 4 charging ports should be set to 0.5A for all 4 channels.
    - It is recommended that staff disconnect batteries if they will not be used for more than 1 month.
- Any damaged or punctured battery should be disposed of safely.
- Never charge, discharge, use, or store a damaged or puffy/swollen lithium polymer battery.
- Please make sure that participants understand that the device contains a cell phone chip and that normal precautions need to be taken e.g., do not get the device wet, do not put the pillbox in a microwave oven or keep close to a hot stove etc.
- Do not charge batteries unless they are at 40% or less.
- Remove the battery from the unit if it will not be used for several months, to avoid low voltage drain on the battery.
- Never leave a charging Lithium Polymer battery pack unattended.
- Request Wisepill provide site training on the battery safety, usage, storage, and charging UNIROSS and EMB batteries.
- Do not charge externally (i.e., using a multi charger)
- Can be charged in the evriMED module with the provided USB cable and a standard USB charger.

HOWELL batteries

- Can be charged externally using a multi charger.
- Can be charged in the evriMED module with the provided USB cable and a standard USB charger.

Multi-Charger:

- If the multi charger has a rotary dial for setting the charging current, set the dial to 0.5 Amp.
- Charge the batteries on a non-flammable surface.
- Do not charge batteries near flammable products or liquids.
- Do not leave unattended.
- After charging, store batteries separately, preferably in the EDM module plastics.
- Keep the batteries in a cool place.

If there are concerns with the battery or EDM device, promptly notify the PHOENIx Clinical Management Committee and Wisepill Representatives.

4.3.5.3 Non-Adherence Episode

Identification of a device with 3 days of no openings within a 7-day period triggers outreach from the site staff (see below).The non-adherence eCRF should be completed once for the full period of the triggered episode (i.e., until device openings are recorded for 5+ doses within a 7-day period). In other words, start with the day on which third dose of 7 is missed and continue until the threshold of 3 out of 7 missed doses no longer applies. See the figure below for an example of a 13-day episode (May 14-26).

Sample dosing history																						
Dose taken	1	1	1	1	0	1	1	0	0	0	1	0	1	1	0	0	0	1	1	1	1	1
Date in May	6	7	8	9	10	11	12	13	14	15	16	17	18	19	20	21	22	23	24	25	26	27
Threshold																						
Dosing days							6	5	4	3	3	3	3	3	3	3	3	3	4	4	4	5
Window period							7	7	7	7	7	7	7	7	7	7	7	7	7	7	7	7
				dose taken																		
				dose missed																		
				= non-adherence episode																		

If the HHC subsequently has 3+ days of no openings within a 7-day period, a new non-adherence form should be completed. More than one reason can be recorded on the form if applicable. Treatment holds per the study do not need to be recorded on this form.
If the data later backfills (e.g., in an area with poor network coverage), the non-adherence eCRF will be disregarded in analysis and does not need to be corrected.

4.3.5.3 Backfilled Data: Under certain conditions (e.g., poor network coverage or a critically low battery), the EDM device will continue to record heartbeat and intake events. The next time that the EDM device can connect to the server these older events will be uploaded and backfilled. The newer/live events will be sent first, and the older events will be sent later in descending order. Because the oldest event was sent last, some fields like the “Last Seen” field will reflect the last event that was reported. This problem will correct itself with the next real-time data transmission. Other fields are accurate.

4.3.6 EDM DATA FEEDBACK AND ADHERENCE COUNSELING

A non-adherence episode will be identified through EDM monitoring; 3 days without an opening from a functioning device in a 7-day period will alert the site staff that outreach is needed. Recall that you will have told participants about this during the education session and have reminded them about it throughout the study. Sites may decide to conduct outreach after 1 or 2 days of non-openings at their discretion and on a case-by-case basis.
Deciding to reach out to a participant earlier (before the occurrence of a full non- adherence episode) may be a helpful decision by site staff who may be able to provide adherence support before a “full” non-adherence episode occurs and the effectiveness of the medication could be compromised. Preventing a non-adherence episode of 3 missed doses in 7 consecutive days would always be preferred from the protocol team, the site, and the participant perspectives. Sites should keep a list of HHCs with known persistent poor network coverage and thus frequent apparent non-adherence

episodes that later backfill. Under these select circumstances, investigation of the episode and completion of the non-adherence eCRF may be delayed until the HHC comes into the site to see if the EDM device data backfills. See section 4.3.5.4 for further information about how poor cellular network coverage may influence data transmission. Each EDM is associated with a unique HHC but contact information for some devices will be shared—for example, contact information for devices linked to a child may be that of the mother. Sites will determine the specific outreach procedures to be used at their site. We recommend starting with a call or text and moving to an in-community visit only when other attempts to connect with the HHC fail. Non-adherence episodes (see section 4.3.5.3) should be documented with the Non-Adherence Episode eCRF (see Section 6.0) which asks sites to enter start and end dates of the episode, details of contact attempts, and reported reasons for the event. If a HHC self-reports non-adherence, complete a Non-Adherence Episode CRF.
The process of outreach should follow a tailored approach. Assess if the non- opening events are behavioral (non-opening by the participant) versus technical (device non-communication with the server). If there is or may be a technical problem, proceed to trouble shooting the device (see the device manual). If the cause was behavioral, (1) explore the situation with the participant (or their parent/guardian), (2) explore participant's (or their parent/guardian) motivation and intentions for daily dosing, (3) create a tailored plan for next steps for this participant, and (4) establish a time for the next contact/follow-up. This outreach can be done over the phone or in person. For repeat behavioral non-adherence episodes that occur between visits, consider addressing second, third and so on episodes in person. When possible, go into the community instead of asking the participant to come to the clinic. Do not assume these non-adherence episodes are unintentional. Let the person tell you what the situation is/was and what they think might help.

4.3.7 ADHERENCE SUPPORT DISCUSSIONS (FACE TO FACE DISCUSSIONS WITH EDM DOSING GRAPHS)

BASIC HH UNIT PROCESS DISCUSSION

1. Welcome and build rapport and engage in site discussion of the adherence goals for the study and the partnership needed to attain them.
2. If a site has opted for some additional incentives to keep adherence high, these can be discussed. Highlighting successful numbers of HHCs, without their names but also at the site, should also be included so that HHCs get the idea that other HHCs are taking their medications as prescribed, even in their own city.
3. Hand out EDM dosing graphs, explain them and allow each member to review their own sheets and sheets of others [this should only be done if the participant gives permission].
4. Facilitate a discussion around what they think of the dosing graphs and whether there are times/situations that people see where dosing seemed to go very well. What characterized those times/situations?
5. Are there non-adherence episodes or periods that were challenging? What characterized those times/situations?
6. Facilitate discussion around desires to address non-adherence episodes and ways to do so.
7. Close with discussing action items and praise/appreciation for ongoing participation in the study. Allow the HHCs to keep their EDM dosing graph and if they desire, to post them at home to show their accomplishments and their most important help with this game-changing research.

4.3.7.1 Facilitated Discussions with HH Unit/Members

At follow-up visits, HH units meet with a designated clinical site staff member to share experiences, discuss how things have been going with taking study medication, and talk over each HHC's EDM dosing graph. The process of this should be participant-centered. Moreover, if non-adherence episodes have been noted by the site, the HH should already have had some outreach by the site and the HH should be expecting some additional support from the site (unless all attempts to reach the HH were unsuccessful). The site approach to supporting adherence should clearly define the basic characteristics of discussions with HH units or unit members. Approaches should specifically avoid negative or punitive approaches to identified non- adherence. Sharing dosing information is meant to foster an open discussion. The process (see Box above) is intended to foster investment and engagement in dosing. A site should feel empowered to have site-appropriate interventions to improve adherence. While the example included here depends heavily on adherence dosing graphs, an intervention can be implemented without dosing graphs, if needed, by asking people to reflect generally on each area. If using graphs, however, be sure to explain the graphs for the first few meetings (pay special attention to literacy and walk members through their dosing graphs specifically for first visit and all subsequent visits for those who do not easily follow the graph). You will likely need to explain them to children throughout the study. When looking over their dosing graphs, members may want to mark them and children may want to draw on them—this is totally acceptable. They can circle, underline and so on while also discussing graphs with each other in terms of where things went well and did not go so well.

Sites can also use a "buddy approach" where HHCs choose a "buddy" for mutual support. This can be a person from the same HH or another HH. If HHs are willing, (especially adolescent HH members), support groups can be formed to support and motivate each other.

4.3.7.2 Special Considerations

When meeting with an individual and not all the members of the HH participating in the study, the same process can be applied. Do not share dosing graphs of HH members who are absent from the meeting but encourage members to share information from the meeting with the absent member. Do not include dosing graphs of absent HHCs to avoid potential social harm, which can happen if the absent member(s) has different patterns of dose taking. If that dosing graph shows far higher or far lower adherence than the rest of the HH members, the meeting can become focused on the absent person rather than those present.

4.3.8 GUIDANCE FOR FACILITATION OF HH UNIT LEVEL DISCUSSIONS

Do NOT underestimate the skills it takes to facilitate group sessions. Practice and pay special attention to the pointers below.

AVOID

- Prolonged focus on one individual
- Rapid close-ended questions
- School-room approach
- Shaming/judging
- Fostering rivalry - use praise and competition very cautiously

PROMOTE

- Questions that engage more than one-unit member
- Discussions between unit members
- Use of "I" statements
- Sharing experiences
- Development of ground rules

DE-ESCALATE

- Normalize challenges
- Watch for the unit "ganging up" on a member and diffuse when needed by redirecting focus
- Violence- watch for gender-based or potential for physical or emotional repercussions of anything discussed - create a safe space but be aware that your ability to maintain this space ends when you leave.

4.4 OPTIMIZING AND EVALUATING THE PERFORMANCE OF THE EDM DEVICE/RAPID PROCESS IMPROVEMENT (RPI)

RPI will be initially used to optimize acceptance and operational performance of the EDM device. These data can be kept in a local database along with the EDM device functionality log. For the first 6-months after each site's opening to enrolment, or until the site determines that there are no issues, the EDM Support Consultant will contact the sites for these data on a monthly basis and report back to sites to resolve identified issues. If the site has issues that need to be addressed right away, they can also contact the EDM Support Consultant at Wisepill Technologies .

4.5 LABELLING OF THE EDM CONTAINER

Wherever there are two or more devices in a HH, the HHCs should be encouraged to decorate or label their own EDM container, to be able to identify their container easily. Children can, for example, draw on the container or put stickers of superheroes on the container. If the sites prefer to provide stickers for the HHC to decorate the container, this can be done.

4.6 DETERMINING AND SUMMARIZING MISSING DOSES

The EDM device will be the primary source of data for determining how many doses have been missed during the 6-month treatment period; these doses may then be given during a treatment extension period. Assessments should be made at each study visit and then summarized at the week 26 study visit as follows:

- For the follow-up period since the last visit, site staff should review with the HHC:
- the adherence graph, and 2) if relevant, any non-adherence episode CRFs. This conversation is designed to adjust the number of missed doses according to the EDM for any instances of technical failures or device non-use. It can likely occur within the already planned counseling sessions as described above. If the participant does not recall his/her dosing history, rely on the EDM device data; it is our primary adherence estimate.
- To optimally frame the conversation, site staff should make a statement such as, "We want to provide every participant with the opportunity to take the full recommended 182 doses during the course of the study, so we are hoping you can help identify the number of doses left for you personally to reach that amount." This type of language will minimize bias in the participant's reporting (e.g., wanting to appear more adherent than they may have been).
- Study staff should indicate the total number of doses missed since the last visit on the Adherence Summary eCRF. In the event of discrepancies in the EDM data and the participant's report, the larger estimate should be recorded. Complete reconciliation of discrepancies is not important, and conflict with the participant should be avoided.
- Pill counts are not required for determining the number of missed doses but may be reviewed by site staff if desired. They may also be used if EDM device data is not available (e.g., in the case of device failure).
- Site staff should review all Adherence Summary eCRFs for the HHC {i.e., Adherence Summary, TB Drugs Adherence Self Report, Non-Adherence at the week 26 visit and calculate the number of missed doses to be given out at that time. A maximum of 8 weeks (i.e., 56 doses) should be given.

4.7 MANAGEMENT OF THE DEVICE DURING STUDY DRUG HOLDS

When the decision has been made to hold the study drug, the site should collect the device from the HHC. To stop triggering of low adherence reports, the intake period should be stopped or the battery disconnected. Note: Data during this period will no longer appear in the adherence reports. See below for silencing the alarm.

Be sure not to end participation unless the HHC is permanently removed from the study. When the study drug hold is over, the intake period should be re-started or the battery reconnected prior to returning the EDM device to the HHC

4.8 TURNING THE ALARM ON/OFF

The alarm can be activated by checking the "*Set as evriMED alarm*" checkbox while creating an intake schedule. The alarm may need to be silenced (e.g., per the participant's request or during a study drug hold). To stop the alarm, sites should delete the current intake schedule, create a new one and ensure that the "*Set as evriMED alarm"* checkbox is unchecked. This switch can take place remotely (i.e., participants do not need to bring the device to clinic). Note that the LED lights will not go off when disabling the alarm via the "*Set as evriMED alarm"* checkbox when creating the intake schedule. Disabling the alarm will disable both the sound and LED lights.

4.9 DECLINED EDM USE

HHCs may still take part in the study if they decline the EDM, although they should be encouraged and supported to try using it. If a HHC permanently declines use of the EDM before week 26 and wishes to continue taking the study drug, sites should do the following:

1. Retrieve the device and "End Participation" for the EDM only on the Wisepill website (i.e., keep the participant in the rest of the study).
2. If a HHC self-reports non-adherence during the remainder of their time taking the study drug, complete a Non-adherence eCRF.

4.10 END OF EDM DEVICE USE

The EDM device should be retrieved from HHCs when they have completed taking study drug and participation is ended on the Wisepill website. The plastic box or bag container for the EDM device can be kept by the HHC. The participant should continue with the rest of study procedures. HHCs may complete their study drug at week 26 or they may extend longer to cover missed doses as indicated in section 4.6. HHCs should continue to use the EDM for any doses taken after week 26. Once all doses are complete, the site needs to document the end of the dosing period
and end participation on the Wisepill website to avoid continued alarms. The EDM device can be brought back to the clinic at a subsequent visit (routinely scheduled or specifically for this purpose at the site's discretion) and documented on the appropriate eCRF. The electronic insert can be inserted into a new container for reuse with other HHCs (see labelling instructions in the Wisepill EDM device user manual).
